# A Systematic Review of the Impact of Remote Working Referenced to the Concept of Work–Life Flow on Physical and Psychological Health

**DOI:** 10.1177/21650799231176397

**Published:** 2023-06-30

**Authors:** John Wells, Florian Scheibein, Leonor Pais, Nuno Rebelo dos Santos, C.- Andreas Dalluege, Jan Philipp Czakert, Rita Berger

**Affiliations:** 1 School of Health Sciences, South East Technological University; 2 Center for Research in Neuropsychology and Cognitive and Behavioral Intervention (CINEICC), Faculty of Psychology and Education Sciences, University of Coimbra; 3 Research Centre in Education and Psychology (CIEP-UÉ), School of Social Sciences, Universidade de Évora; 4 Institut für Betriebsanalyse und Kommunikationsforschung; 5 Facultat de Psicologia, Universitat de Barcelona

**Keywords:** remote working, health impacts, work–life flow, occupational health nursing, practice

## Abstract

**Background:**

COVID-19 accelerated the adoption of remote working in which employers’ obligations for employees’ health and well-being extended into the home. This paper reports on a systematic review of the health impacts of remote working within the context of COVID-19 and discusses the implications of these impacts for the future role of the occupational health nurse.

**Method:**

The review protocol was registered with PROSPERO (CRD42021258517) and followed the PRISMA guidelines. The review covered 2020-2021 to capture empirical studies of remote working during the COVID-19 pandemic, their physical and psychological impacts and mediating factors.

**Results:**

Eight hundred and thirty articles were identified. After applying the inclusion criteria, a total of 34 studies were reviewed. Most studies showed low to very low strength of evidence using the GRADE approach. A minority of studies had high strength of evidence. These focused on the reduced risk of infection and negative effects in terms of reduced physical activity, increased sedentary activity, and increased screen time.

**Conclusion/Application to Practice:**

The synergy of work and personal well-being with the accelerated expansion of remote working suggests a more active role in the lives of workers within the home setting on the part of occupational health nurses. That role relates to how employees organize their relationship to work and home life, promoting positive lifestyles while mitigating adverse impacts of remote working on personal well-being.

## Background

COVID-19 has had major impacts on population and individual health (Solmi et al., 2022) and a transformational impact on the widespread adoption of remote working by both employers and employees (Ng et al., 2021; Vyas & Butakhieo, 2021). Remote working includes what is known as telework and working from home (WFH). The European Framework Agreement on Telework (2006) defines telework as a “form of organizing and/or performing work, using information technology, in the context of an employment contract/relationship, where work, which could also be performed at the employer’s premises, is carried out away from those premises on a regular basis” (Gabaglio et al., 2002). WFH may be defined as a situation in which an employee works mainly from home and usually communicates with their employer and co-workers by digital means (e.g., email, video conferencing, mobile phone).

WFH is predicted to accelerate over the coming years as employees become more focused on the interface of quality of life with that of work–life (Pheng & Chua, 2019; Philips, 2020) and employers see the cost-efficiency benefits of locating employees at home (Parker, 2020). Governments from a number of European countries agreed on the adoption of the EU framework on telework (European Social Partners ETUC (2006). Consequently, countries such as Ireland are enshrining into employment law a right to work from home if an employee can demonstrate that doing so would have no material negative impact on the employer (Department of Enterprise, Trade and Employment, 2021).

Such a significant shift in the locus of employment may change the nature of health and well-being issues for many workers (Oakman et al., 2020); the obligations of employers to support worker well-being within the home (Philips, 2020) and, by implication, the future practice of occupational health nurses as this relates to both positive and negative health impacts on workers (Gleason, 2021). Remote work may affect both physical and psychological health ranging from musculoskeletal disorders, fatigue, and stress (Collins et al., 2016; Oakman et al., 2020). Alternatively, there is evidence that it can have positive social and psychological benefits by improving personal mood (Anderson et al., 2015).

Literature indicates gendered differences in reported negative health impacts on women compared with men when forced to work from home (Anderson & Kelliher, 2020). Such findings suggest a need for consideration of gendered differentials as to the nature of health interventions where the home is the employee’s working environment.

Linked to these impacts is the adoption of the concept of decent work in the workplace (dos Santos, 2019; Ferraro et al., 2018). The 8th Sustainable Development Goal of the 2030 United Nations Agenda includes the adoption of decent work principles to reinforce employer commitment to health and safety at work (Ferraro et al., 2015, 2018; International Labour Organization (ILO), 2012; dos Santos, 2019).

Such international pronouncements combined with rapid changes to the workspace suggest that both current and future adoption of remote working, combined with the personal aspirations of workers for better quality of life (Sull et al., 2022), will require employers to demonstrate commitment to worker well-being. In many organizations, occupational health nurses as the operational arm of such commitment will need to master and engage with an interactive complexity of health promotion, personal workspace safety, and quality life issues that previously, it could be argued, were not in their purview.

This systematic review reports on health impacts of remote working within the context of WFH as happened in many countries during the height of the COVID-19 pandemic between 2020 and 2021. The review is referenced to trends in remote work, the concept of work–life flow (WLF) rather than work/life balance, and how trends and new concepts may change the practice paradigm of occupational health nursing in the future.

### The Concept of Work–Life Flow

There is a significant relationship between an employee’s psychological health and organizational success (Grawitch et al., 2006; Loon et al., 2019; World Health Organization [WHO], 2001). There are some tensions between worker well-being and organizational outcomes that suggest that aligning employee self-interest with organizational goals is in the interests of the employer (Grawitch et al., 2006; Loon et al., 2019). Embracing decent work as an operational framework to promote and support optimal positive employee health and protection as this relates to personal aspirations, work demands and work enjoyment reflective of Goal 8 of the UN agenda for Sustainable Development (United Nations (UN), 2015) achieves this.

Optimizing workers’ well-being within the home-based workplace has been conceptualized as WLF, sometimes called Work–Life Integration. This conceptualizes that it is impossible to separate/compartmentalize one’s personal life from one’s working life as they have an iterative relationship. WLF differs from Work–Life Balance in that the latter separates work from personal life to promote a boundary-enforced evenness between the two (Guest, 2002), which, in the context of WFH, can be difficult.

Czakert et al. (2022), state that WLF has several features: It adopts the resource–demand-based theory where both resources and demands can stem from work or non-work, personal or environmental domains; aims toward a dynamic balance between resources and demands; promotes this balance being weighted in favor of positive challenge; recognizes that subjective experiences arise from interaction between people and their environments; recognizes that periods of lower challenge or rest are necessary to sustain optimal functioning and acknowledges that meaningfulness is a critical job resource to maintain the optimal skill-challenge balance over time.

The impetus for the adoption of WFH means that the physical boundary between an employee’s work and personal space is synergized. WLF assuming a relationship between the two (van Zoonen et al., 2020), promotes holistic employee well-being through a focus on the interface between work and personal life. WLF, therefore, suggests an expansive role for the occupational health nurse in supporting employees’ well-being in relation to this interface compared with the boundary enforcement implied in Work–Life Balance.

### The Role and Practice of Occupational Health Nursing

In 2001, the World Health Organization identified that changes in the workplace and public expectations with regard to quality of working life meant that the role of occupational health nurses would need to become more expansive (WHO, 2001). Occupational health nurses’ roles encompass environmental and risk management, health and safety advice, assessment as this relates to human resource management and development of employee well-being programs as part of a business strategy (American Board for Occupational Health Nurses, 2021).

Recently, because of COVID-19, the occupational health nurse’s role has come to the fore in terms of return to work and in terms of their future role as it relates to vaccination and assessment of employees’ health readiness to be at work, whether WFH or at the employer’s physical location. This suggests that it would be worthwhile to explore what health issues arise for remote workers and how they can be addressed by occupational health nurses in current and future remote working environments.

## Method

The question posed for this review was “What is the impact of remote work on individuals’ physical and psychological health?” The review protocol was registered with PROSPERO (CRD42021258517) and followed the PRISMA guidelines.

Studies exploring physical and psychological impacts of WFH that result from sustained exposure to remote working and mediating factors were included in this review. Physical impacts included musculoskeletal disorders, back pain, neck pain, repetitive strain injury, eye strain, vision, fatigue, and sleep. Psychological impacts included substance use, online behavior, depression, anxiety, stress, mental health, mental illness, sense of well-being, and fatigue.

A systematic search of the literature was conducted utilizing a series of search terms generated through initial reading of relevant literature (see Table 1). Articles were reviewed from ClNAHL, MEDLINE, APA PsycINFO, and APA PsycArticles. Search terms related to remote working (a) and mental impacts (b) or physical impacts (c) or COVID-19 (d). A timeframe between 2020 and 2021 was selected in recognition of the dramatic change in work practices wrought by COVID-19 and the resulting policy responses.

**Table 1.** Search Terms

1. “remote work “OR “virtual work “OR telework OR “work from home” AND 2. depression OR anxiety OR stress OR “mental health “OR “mental illness “OR distress 3. “psychological impacts “OR well-being ORwell-beingOR substance use OR addiction OR “internet use “OR gaming OR gambling2.“musculoskeletal disorder*”OR “back pain” OR “neck pain” OR “repetitive strain injury”OR “eye strain” OR vision OR fatigue OR sleep 4. COVID OR COVID-19 OR “corona virus” OR pandemic

All included studies were in the English language. Reviews, commentaries, single-case reflections and studies not focussing on the working population were excluded. The strength of evidence was evaluated using the GRADE approach.

## Results

**Figure 1.**
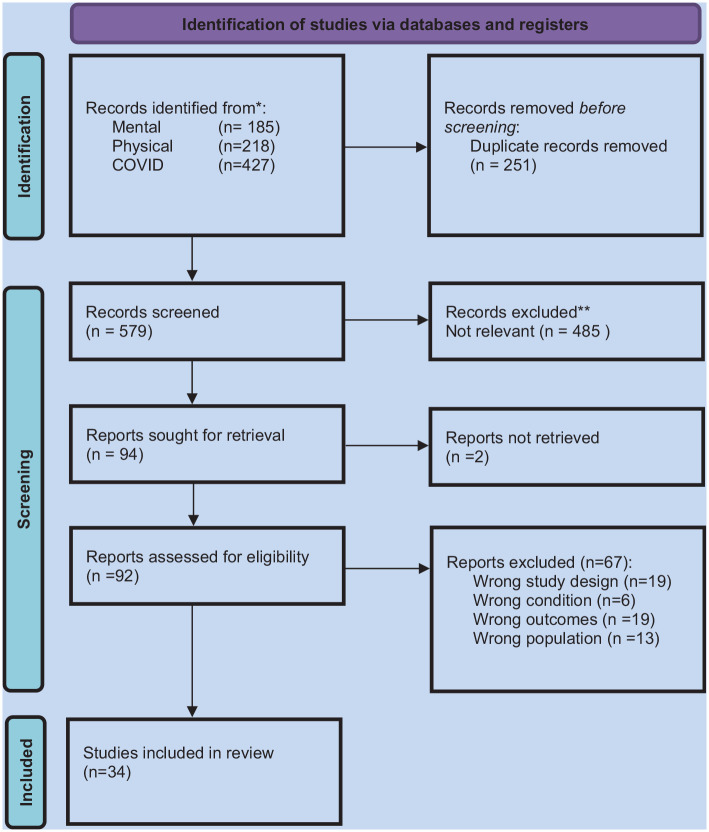
PRISMA Flowchart

Eight hundred and thirty articles were identified through the literature search. Two hundred and fifty duplicates were removed, leaving 579 articles for screening. Four hundred and eighty-five articles were removed due to non-relevance, leaving 94 records sought for retrieval (of which two could not be retrieved). Ninety-two were screened for eligibility leading to the exclusion of 58 articles for the following reasons: wrong study design (*n* = 19); wrong condition (*n* = 6); wrong outcomes (*n* = 19), and wrong population *(n* = 13). This left 34 articles for inclusion in the review (see Figure 1). The retrieved studies are described in more detail in Table 2: Study Description.

**Table 2.** Study Description

Author (year)	Primary outcomes	Study population (*n* = sample size)	Country	Study type
Ahmed et al. (2020)	Work attendance during the first 3 days after onset of acute respiratory illness	People with access to remote working (*n* = 198)People without access (*n* = 1,164)	United States	Cross-sectional, survey
Astell-Burt, & Feng (2021)	Respite, connection, and exercise and exercise during the COVID-19 pandemic	General population (*n* = 3,043)	Australia	Cross-sectional, survey
Bennett et al. (2021)	Prevalence and nature of video conferencing fatigue	Remote workers (*n* = 55)	United States	Cross-sectional, survey
Björndell & Premberg (2021)	Physicians’ experiences of video consultation with new patients visiting a publicly owned virtual primary care clinic	Primary care physician remote workers (*n* = 10)	Sweden	Cross-sectional, interviews
Chang et al. (2021)	Relationship between proactive coping, future time orientation, and perceived work productivity during the coronavirus (COVID-19) pandemic	Remote workers (*n* = 778)	Taiwan and United States	Longitudinal, field experiment
Cheng & Zang (2021)	Relationship between COVID-19 task setbacks and exhaustion; influence of task interdependence	Fulltime teleworkers due to the pandemic lockdown (*n* = 1,022)	United States	Longitudinal, diary
Darouei & Pluut (2021)	Influence of working from home on experiences of time pressure, work–family conflict, and work-related employee well-being	Professional workers (*n* = 34)	The Netherlands	Longitudinal, survey
Dhont et al. (2020)	Impact of social isolation and working from home and to guide future work.	Remote workers physicians (*n* = 43)	Italy	Cross-sectional, survey
Edwards et al. (2021)	PM2.5 exposure after lockdown	Embassy workers (*n* = 4)	Nepal	Cross-sectional, air monitors
Estrada-Muñoz et al. (2021)	Levels of technostress	Teachers (*n* = 3,006)	Chile	Cross-sectional, survey
Fischer et al. (2020)	Impact of telework on risk for SARS-CoV-2 infection	Case patients (*n* = 153); Control (*n* = 161)	United States	Longitudinal, case-control
Fukushima et al. (2021)	Comparison of physical activity (PA) and sedentary behavior (SB) levels during work time between those who work from home (WFH) and at workplaces (no WFH), and by WFH subgroups.	General population (*n* = 1,239)	Japan	Cross-sectional, surveys
Giovanis & Ozdamar (2022)	Impact of WFH on the individuals’ perception about their future financial situation and their mental well-being	General population (*n* = approx. 30,000)	Turkey	Cross-sectional, survey
Grech et al. (2022)	Increase of back pain complaints has increased from pre-COVID-19 to during the COVID-19 period	General population (*n* = 388)	Malta	Cross-sectional, survey
Hallman et al. (2021)	Extent to which the 24-hour allocation of time to different physical behaviors changes between days working at the office (WAO) and days WFH in office workers during the pandemic.	Workers working remotely and in office (*n* = 27)	Sweden	Longitudinal, accelerometer, and diary
Hoffman (2021)	Impact of companion animals on well-being for those teleworking	Individuals who only had dogs (*n* = 90), who only had cats (*n* = 90), who had both dogs and cats (*n* = 50), and who had neither dogs nor cats (*n* = 150)	United States	Cross-sectional, survey
Houle et al. (2021)	Telecommuting and individual associated factors related to headache and neck pain occurrence in telecommuters over a 5-day follow-up.	Telecommuters (*n* = 162)	Canada	Longitudinal, emails, surveys
Ng et al. (2021)	Effect of work overload (workload and techno overload), on behavioral stress,	Remote workers (*n* = 530)	Italy	Cross-sectional, survey
Izdebski & Mazur (2021)	Relationship between occupational activity and mental health during the first COVID-19 lockdown	General population (*n* = 3,000)	Poland	Cross-sectional, social media data
Kawashima et al. (2021)	Relationship between telework implementation and the presence of a fever (body temperature higher than 37.5 °C) within 1 month as a surrogate indicator of COVID-19 infection	Social networking service users general population (*n* = 270,000)	Japan	Cross-sectional, social media data
Kenny (2020)	Prevalence, incidence, characteristics, and impact of self-perceived dysphonia and vocal tract discomfort in those working from home during COVID-19	Remote workers (*n* = 1,575)	Ireland	Cross-sectional, survey
Kumar et al. (2020)	Perceived stress scores (PSS) and COVID-19-related stress (COVID-SS) scores to evaluate general and COVID-19-induced stress	Academic staff (*n* = 9)	United States	Cross-sectional, case study
Lafferty et al. (2022)	Impact of the COVID-19 pandemic on employment and care-giving responsibilities	Family carers (*n* = 16)	Ireland	Cross-sectional, interviews
Larrea-Araujo et al. (2021)	Ergonomic risk factors for teleworking	Researchers (*n* = 204)	Equador	Cross-sectional, survey
Limbers et al. (2020)	Associations between parenting stress, quality of life, and physical activity	Full-time working mothers (*n* = 200)	United States	Cross-sectional, survey
McDowell et al. (2020)	Associations of changing COVID-19-related employment conditions with physical activity and sedentary behavior	General population (*n* = 2,303)	Ireland	Cross-sectional, survey
Oksanen et al. (2021)	Potential stress effects of social media communication (SMC) at work	General employees before COVID crisis (*n* = 1,308) and general employees during COVID crisis (*n* = 1,081)	Finland	longitudinal, social media data
Reizer et al. (2021)	Links between conditions of uncertainty with psychological distress during the coronavirus 2019 lockdown.	Married parents working from home (*n* = 186)	Israel	Cross-sectional, survey
Rohwer et al. (2020)	Insights into job demands and resources in virtual teamwork	Virtual team members (*n* = 46)	Germany	Cross-sectional, survey
Shklarski et al. (2021)	Significant challenges and specific adaptations to this experience of providing remote therapy from home during the COVID-19 pandemic	Remote working therapists (*n* = 92)	United States	Cross-sectional, survey, and interviews
Shockley et al. (2021)	Impact of camera on videoconferencing fatigue	Remote working healthcare workers (*n* = 103)	United States	Longitudinal, field experiments
Tušl et al. (2021)	Actual and perceived overall impact of the COVID-19 crisis on work and private life, and the consequences for mental well-being (MWB), and self-rated health (SRH)	German and Swiss employees’ general population (*n* = 2,118)	German and Switzerland	Cross-sectional, survey
van Niekerk & van Gent (2021)	Mental health and well-being	University staff members (*n* = 280)	South Africa	Cross-sectional, survey
Wang et al. (2020)	Challenges experienced by remote workers at this time	Remote workers (*n* = 561)	China	Cross-sectional, interviews, and survey

Most studies originated in Europe (*n* = 14) followed by North America (*n* = 9); Asia (*n* = 6); South America (*n* = 2); Africa (*n* = 1); Australia (*n* = 1) and one inter-continental study (*n* = 1). In studies where population size was reported the size ranged from 4 (Edwards et al., 2021) to 270,000 (Kawashima et al., 2021). Studied populations included remote workers (*n* = 8); the general population—this included a subset of remote working people (*n* = 7); general employees—this included a subset of remote working people (*n* = 5); remote working healthcare workers (*n* = 4); remote working academic and education staff (*n* = 4); remote working parents and/or carers (*n* = 3); remote working embassy staff (*n* = 1); pet and non-pet owning workers (*n* = 1); and social media users (*n* = 1).

Most of the studies used a cross-sectional research design (*n* = 26) while a minority of studies used a longitudinal design (*n* = 8). A majority of cross-sectional studies utilized a survey approach (*n* = 18) while other approaches included interviews (*n* = 2); interviews and surveys (*n* = 2); social media data (*n* = 2) and the use of an air monitor to test exposure to air pollutants in the home compared with the outside environment (*n* = 1). Longitudinal studies included those that adopted a quasi-experimental design (*n* = 1); social media data (*n* = 1); emails and survey (*n* = 1); accelerometer and diary (*n* = 1); survey (*n* = 1), and diary (*n* = 1).

### Quality Appraisal

Most of the main findings of the retrieved studies could be ranked as low to very low-quality certainty of evidence due to a range of reasons including small sample sizes, a lack of agreement in how to measure what constitutes WFH, and the lack of reporting of significant statistical effect sizes. A summary of findings can be found in Table 3. Only a minority were found to have high certainty of evidence. These included studies finding a relationship between WFH and protection against COVID-19 (Fischer et al., 2020); reductions in physical activity and increased sedentary time (Fukushima et al., 2021) and increased sitting and screen time (McDowell et al., 2020).

**Table 3.** GRADE Table

Author, year	Key relevant findings	Certainty of evidence
Ahmed et al. (2020)	WFH associated with less smoking	Low**
	WFH associated with increased number of people reporting excellent health	Low**
	WFH associated with less reporting of conditions associated with influenza	Low**
Astell-Burt & Feng (2021)	WFH associated with increased exercise. Geography and enjoyment of outdoor spaces being mediators.	Low**
	WFH associated with increased enjoyment of outdoor spaces	Low**
Bennett et al. (2021)	WFH associated increased videoconferencing fatigue	Low**
	Switching off microphone associated with decreased videoconferencing fatigue	Moderate***
	Increased perceived group belongness associated with decreased videoconferencing fatigue	Moderate***
Björndell & Premberg (2021)	WFH associated with increased perception of working in peace	Very low*
	WFH associated with less stress	Very low*
	WFH associated with increased enjoyment of the home	Very low*
Chang et al. (2021)	In the WFH context, proactive coping is associated with self-perceived productivity	Low**
	In the WFH context, proactive coping is associated with future time orientation	Low**
	In the WFH context, future time orientation is associated with self-perceived productivity	Low**
Cheng & Zang (2021)	In the WFH context, higher task interdependence is negatively associated with emotional exhaustion	Low**
Darouei & Pluut (2021)	WFH associated with decreased time pressure associated with less family conflicts	Low**
Dhont et al. (2020)	WFH associated with less depression	Very low*
	WFH associated with less guilt	Very low*
Edwards et al. (2021)	WFH associated with decreased exposure to air contaminants	Very low*
Estrada-Muñoz et al. (2021)	WFH associated with increased “technoanxiety”	Very low*
	WFH associated with increased “technostrain”	Very low*
	WFH associated with increased “technofatigue”	Very low*
Fischer et al. (2020)	WFH may reduce risk of COVID-19 infection	High****
Fukushima et al. (2021)	WFH associated with reduced physical activity	High****
	WFH associated with increased sedentary behavior	High****
Giovanis & Ozdamar (2022)	WFH associated with decreased mental health as measured by the General Health Questionaire (GHQ)	Moderate***
Grech et al. (2022)	WFH associated with increased back pain	Very low*
Hallman et al. (2021)	WFH associated with increased sleep	Moderate***
	Increased sleep associated with decreased work time	Moderate***
	Increased sleep associated with decreased leisure time	Moderate***
Hoffman (2021)	Pet dogs associated with increased socialization with other people	Very low*
	Pet dogs associated with healthy amounts of physical activity	Very low*
	Pet dogs associated with taking at least one 15-minute walk during the workday	Very low*
Houle et al. (2021)	Headache related disability associated with increased risk of headaches	Low**
	Neck pain related disabiluity associated with increased risk of neck pain	Low**
Ng et al. (2021)	Workload associated with increased stress	Moderate**
	Job crafting associated with less stress	Moderate**
Kawashima et al. (2021)	WFH associated with lower fever rates	Low**
Izdebski & Mazur (2021)	Female gender associated with decreased mental health	Low**
	Threat of worsening employment terms associated with decreased mental health	Low**
Kenny (2020)	Frequently raising/straining voice (increased frequency) increased risk of new onset dysphonia	Moderate**
	Older age associated with increased risk of new onset dysphonia	Moderate**
	Poor air quality associated with increased risk of new onset dysphonia	Moderate**
	Increased stress associated with increased risk of new onset dysphonia	Moderate**
	Higher frequency associated with self-rated dysphonia severity	Low**
	Poor posture associated with self-rated dysphonia severity	Low**
	Raising or straining voices associated with increased risk of new onset vocal tract discomfort	Moderate**
	Increased telephone use associated with increased risk of new onset vocal tract discomfort	Low**
	Increased frequency of frequently raising/straining voice associated with increased risk new onset vocal tract discomfort	Low**
	Increased stress associated with increased risk of new onset vocal tract discomfort	Low**
	Poor air quality associated with increased risk of new onset vocal tract discomfort	Low**
Kumar et al. (2020)	Intervention reduced Perceived Stress Scores	Low**
	Intervention reduced COVID-19-related stress scores	Low**
Lafferty et al. (2022)	COVID-related work challenges associated with increased stress	Very low*
Larrea-Araujo et al. (2021)	Age associated with increased risk of neck ailments	Very low*
	Arm ailments associated with increased risk of forearm	Very low*
Limbers et al. (2020)	Parenting stress associated with a lower quality of life	Low**
	Parenting stress associated with lower social relationships	Low**
	Parenting stress associated with lower environmental quality life	Low**
	Attenuation of negative effect of parenting stress on social relationships and environmental quality of life	Low**
McDowell et al. (2020)	WFH associated with increased sitting time	High****
	WFH associated with increased screen time	High****
Oksanen et al. (2021)	Previous experience using social media communication associated with a decrease in “technostress”	Moderate***
	Previous experience using social media communication associated with a decrease in work exhaustion	Moderate***
	Increase in use of formal social media associated with increased technostress	Moderate***
Reizer et al. (2021)	Intolerance of uncertainty and distress	Low**
Rohwer et al. (2020)	Degree of virtuality associated with boundarylessness	Low**
	Psychological detachment associated with improved sleep quality	Low**
	Perceived stress associated with reduced sleep quality	Low**
Shklarski et al. (2021)	WFH associated with videoconferencing fatigue	Very low**
Shockley et al. (2021)	Having camera on associated with increased videoconferencing fatigue	Moderate***
Tušl et al. (2021)	WFH particularly if experienced for the first time associated with perceived positive impact on work–life	Moderate***
van Niekerk & van Gent (2021)	Psychological distress and mental well-being (MWB)	Very low*
	Age associated with decreased psychological distress	Very low*
	Age associated with increased mental wellbeing	Very low*
	Female gender associated with increased psychological distress	Very low*
Wang et al. (2020)	Ineffective communication associated with work–home interference	Very low*
	procrastination (e.g. social media, long breaks) associated with reduced productivity	Very low*
	Job autonomy associated with increased loneliness	Very low*
	High workload associated with increased devotion to work	Very low*
	Low workload associated with decreased work–home balance	Very low*
	Low social support associated with increased procrastination	Very low*
	High workload associated with increased work–home interference	Moderate***
	High workload associated with increased work–home interference	Moderate***
	Work–home interference associated with increased emotional exhaustion	Moderate***
	How to work interference associated with increased emotional exhaustion	Moderate***
	Loneliness associated with increased emotional exhaustion	Moderate***
	WFH associated with increased loneliness	Moderate***

WFH = work from home.

### Acceleration of Remote Working Practices and Use of Environments

The COVID-19 pandemic dramatically accelerated remote working practices among a range of employees including education and research staff (van Niekerk & van Gent, 2021), healthcare workers (Björndell & Premberg, 2021; Dhont et al., 2020) and family carers (Lafferty et al., 2022). This necessitated the use of non-traditional work environments including bedrooms and living rooms for remote working (Larrea-Araujo et al., 2021) as well as the learning of new skills including how to use new online tools (Björndell & Premberg, 2021) and mitigating the impact of these tools in terms of physical and psychological impacts (Bennett et al., 2021; Shockley et al., 2021).

### Physical Impact

Employees engaging in WFH may lack properly designed ergonomic work environments and suffer from issues such as excess noise, lack of lighting, excess heat, and a lack of adequate furniture (Larrea-Araujo et al., 2021). In this context, a range of physical health complaints including back pain, neck pain, headaches, voice, and vocal tract discomfort were reported (Houle et al., 2021; Kenny, 2020; Larrea-Araujo et al., 2021). In contrast, WFH may also reduce negative health exposures such as infections and exposure to pollution (Ahmed et al., 2020; Edwards et al., 2021; Kawashima et al., 2021).

In a study of researchers by Larrea-Araujo et al. (2021), it was found that after several months of WFH during the pandemic, most study participants reported sensations of tension in the back, lower back, and neck and a third reported sensations of tension in the arm, forearm, hand, wrist and shoulders. The highest incidence is related to the neck and back at the lumbar level.

Such injuries are consistent with physical impacts related to the use of video display terminals and include afflictions such as cervical pain, back pain, herniated disks, sciatica, and disk protrusion. Grech et al. (2022) also found most of those reporting back pain since the beginning of the pandemic were WFH (51.83%; *p* = .01).

Increased use of communication tools was found to result in an increase of diseases of the throat. In a population of remote workers, Kenny (2020) found prevalence rates of 33% and 68% for dysphonia and vocal tract discomfort, respectively. Perceived dysphonia severity was mild in a majority (72%) of cases with dry throat being the most common vocal tract discomfort symptom reported (66%).

Remote working can have positive physical impacts in relation to exposure reduction to common pathogens due to reduced social contacts, resulting in lower reported fever rates (Kawashima et al., 2021) and influenza symptoms (Ahmed et al., 2020). Edwards et al. (2021) also reported lower exposure to air pollution (as measured by particulate matter) in a population of diplomats forced to work remotely due to the pandemic.

WFH does not appear to have an impact on headache and neck pain (Houle et al., 2021). Houle et al. (2021) found that in a population of remote workers future headaches were associated with Headache Impact Test 6 scores (OR (95% CI) = 1.094 (1.042 to 1.148; *R*^2^ = 0.094; *p* < .001) and future neck pain was associated with Neck Bournemouth Questionnaire score (Houle et al., 2021).

### Psychological Impacts

Several positive psychological impacts of WFH were reported. Björndell and Premberg (2021) reported that a population of doctors who moved to remote working felt they could work in peace, felt less stressed, and enjoyed being at home. Darouei and Pluut (2021) found that workers who engaged in WFH were less time pressured and, in turn, this was associated with lower levels of work–family conflict during the working day. Work–family conflict was also found to predict individuals’ next morning engagement, exhaustion levels, and affective states toward the organization leading the authors to recommend that organizations encourage a WFH protocol to protect employee well-being (Darouei and Pluut, 2021).

Tušl et al. (2021) reported that WFH, particularly when experienced for the first time, was generally associated with a positive impact on work–life. Oksanen et al. (2021) found a decrease in workplace exhaustion. In contrast, Dhont et al. (2020) found that people having to work full-time in offices showed higher depressive symptoms than those engaging in WFH. WFH may also lead to increased stress in some instances (Giovanis & Ozdamar, 2022; Kumar et al., 2020). For example, Kumar et al. (2020) found that WFH negatively affected the productivity of researchers engaging in basic science research which led to stress and anxiety. These negative impacts were, however, partially mitigated by engaging researchers virtually in various components of the research planning and preparation of their research (Kumar et al., 2020). Giovanis and Ozdamar (2022) found that WFH negatively affected well-being. However, when workers engaged in a mixed model of WFH and working in the employers’ premises they did not find a negative impact on well-being.

In the context of the COVID-19 pandemic, Tušl et al. (2021) found that about 30% of employees reported that their work and private life had deteriorated and about 10% reported improvements in work–life and 13% reported improvements in private life. van Niekerk and van Gent (2021) found that 27.6% of surveyed staff reported psychological distress with COVID-19-related socioeconomic collapse, contracting the virus, and the completion of the academic year as the respondents’ biggest worries. Estrada-Muñoz et al. (2021) found that teachers who had moved to remote work showed a high level of techno-anxiety (11%) and techno-fatigue (7.2%) with 6.8% surveyed teachers were found to be techno-stressed.

Work demands such as mandatory short-time work or video conferencing were found to have psychological impacts. Mandatory short-time work was strongly associated with perceived negative impacts on work–life (Tušl et al., 2021). WFH was also found to increase videoconferencing fatigue (Shklarski et al., 2021; Shockley et al., 2021).

### Health Behaviors and Mediating Factors

WFH was found to have an influence on time spent sitting and physical inactivity. Fukushima et al (2021) found that reported sitting time in the WFH group was significantly longer than in the non-WFH group (335.7 vs. 224.7 min [74% vs. 50%]) and highest in the maximalist WFH group (WFH 76%–100%). Fukushima et al (2021) also reported that significantly shorter standing/light intensity physical activity was found in the WFH group than in the non-WFH group (LPA, 59.6 vs. 122.9 min [14% vs. 29%] and shorter reported standing/light intensity physical activity and engaging in heavy labor (moderate-to-vigorous PA) were observed in the highest WFH group as measured by the Work-related Physical Activity Questionnaire.

In contrast, Astell-Burt and Feng (2021) found increased physical activity among remote workers through access and appreciation of green and blue spaces. Increasing financial difficulty was associated with lower use of these spaces and less perceived benefit in terms of social connection. Hoffman (2021) found that while exercise was lower in the WFH group, those with pets were more likely to exercise. Hallman et al. (2021), however, did not find any significant changes in reported sedentary, standing, and moving behaviors when comparing WFH and non-WFH days.

Grech et al.’s (2021) results suggest a potential relationship between WFH, back pain, sitting, and not performing physical activity. They found a relationship between WFH and back pain since the onset of the pandemic (*p* = .01) with other risk factors including increases in weight (*p* = .01); sitting most of the time (*p* ≤ .01); not performing any physical activity (*p* ≤ .01), and spending most of their time confined to their homes (*p* = .02; Grech et al., 2021).

Parenting stress and moderate intensity physical activity was associated with social relationships quality of life and environment quality of life (*p* < .05) of working mothers (Limbers et al., 2020). Those working mothers who engaged in higher levels of moderate intensity physical activity had lower negative parenting stress effect on maternal social relationships and environmental quality of life (Limbers et al., 2020).

Sleep was found to play a role in WFH in a number of studies (Hallman et al., 2021; Rhower et al., 2020). Hallman et al. (2021) found that, on WFH days, a population spent more time sleeping than during non-WFH days with a large effect size (*F* = 7.4; *p* = .01; *ηp*^2^ = .22). This increase occurred at the expense of a reduction in work and leisure time by 26 and 7 minutes, respectively. In another study, Rohwer et al. (2020) found that virtual team members reported higher levels of psychological detachment from work, and the level of psychological detachment was associated with better sleep quality butt a higher degree of virtuality was also linked with higher levels of boundarylessness which, in turn, was associated with lower levels of psychological detachment.

A range of human resources and management processes were found to affect outcomes. Chong (2020) found that exhausted employees could draw from external resources (i.e., organizational telework task support) for replenishment. Similarly, Dhont et al. (2021) found that the presence of a supportive institutional program was the only significant factor associated with reported anxiety and depressive symptoms. Ingusci et al. (2021) found that perceived job crafting was a protective factor in mitigating the negative effect of workload and heavy remote working on individual outcomes. In another study (Wang et al., 2021), perceived job autonomy negatively correlated with loneliness and higher workload and monitoring was associated with higher work-home interference but higher workload was also linked to lower procrastination. Self-discipline was a significant moderator of several of these relationships (Wang et al., 2021). Perceived intolerance of uncertainty was linked to psychological distress in a relationship moderated with optimism and work schedule (Reizer et al. (2021). Chang et al. (2021) found a relationship between proactive coping and perceived work productivity; future time orientation—where one’s anticipated future is integrated into the present—was found to be a full mediator in Taiwan and a partial mediator in the United States of this relationship.

Oksanen et al. (2021) found that formal social media communication increased during the pandemic and predicted higher techno-stress. However, techno-stress and work exhaustion decreased among workers already accustomed to using social media communication at work before the crisis. Kenny (2020) found that increasing telecommunication use was associated with worse dysphonia and vocal tract discomfort and that raising or straining the voice while working predicted new onset dysphonia and vocal tract discomfort.

Two studies reported that the method in which videoconferencing technology was used could predict video conferencing fatigue (Bennett et al., 2021; Shockley et al., 2021). The number of meetings and the time spent in meetings was not associated with fatigue but switching off the camera, switching off the microphone and perceived group belongness were associated with protective effects against fatigue (Bennett et al., 2021; Shockley et al., 2021). Several demographic variables were associated with health outcomes including age, gender, and job role. Estrada-Muñoz et al. (2021) found higher fatigue and anxiety factors for female teachers. van Niekerk and van Gent (2021) found that female staff members, staff members with comorbidities, and workers in the administration and service sections were significantly more likely to report psychological distress. However, age was negatively correlated with psychological distress (*r* = .130) and positively correlated with mental well-being (*r* =.153). Shockley et al. (2021) found that women and new employees were particularly prone to video fatigue. Larrea-Araujo et al. (2021) found a link between neck ailments and age.

Remote working ergonomics can be a predictor for a number of ailments. Larrea-Araujo et al. (2021) identified the lack of an ergonomic office chair and working in one’s bedroom as key risk factors for ailments. The authors reported that lack of an ergonomic chair was a key driver of discomfort at the lumbar level of the back and neck and working in the bedroom or dining room as a driver of hand or wrist discomfort Larrea-Araujo et al. (2021).

The COVID-19 pandemic, the perception thereof, and resulting behavior changes were also reportedly associated with significant impact on work and home life. Tušl et al. (2021) found that younger age, living alone, reduction in leisure time, and changes in quantity of caring duties were associated with perceived negative impact on personal life. However, living with a partner or family, short-time work, and increases in leisure time and caring duties was found to have a positive impact. A perceived negative effect of the crisis on work and private life and mandatory short-term work was associated with decreased mental well-being and self-rated health while a perceived positive impact on private life and increases in leisure time were associated with higher reported mental well-being (Tusl et al., 2021). COVID-related changes in terms of increased sedentary behavior may have increased the risk of sedentary-related back pain (Grech et al., 2022).

## Discussion

This systematic review aimed to answer the question what is the impact of remote work on individuals’ physical and psychological health? A secondary aim was to consider the relevance of these findings to the future practice of occupational health nursing. Overall, the quality of the literature was mixed in terms of design and sample size and this needs to be considered when forming a general view in relation to both questions (see “Appraisal” section and Table 3 in relation to the quality of the literature). Consequently, the quality of the literature would suggest that more robust research is warranted. It may also be suggested that such research should be led by occupational health nurses since they will have a pivotal role in any successful support of workers in a WFH setting. With these caveats in mind, the review identified a complex relationship between negative impacts of remote working on specific areas of physical and psychological health, while also highlighting some positive benefits to overall well-being. The implications of these findings for the practice of occupational health nursing are two-fold—first, that practice will need to extend beyond the traditional scope of the working space and, second, training for occupational health nurses will need to encompass a more comprehensive yet nuanced consideration of the iterative nature of workers’ lives with their personal space and concerns when formulating advice and interventions.

It is apparent that negative impacts of remote working are distributed unequally related to age, gender, job role, and whether choice was a determinant in WFH (Estrada-Muñoz et al., 2021; Larrea-Araujo et al., 2021; Oksanen et al., 2021; Shockley et al., 2021; Tusl et al., 2020; van Niekerk and van Gent, 2021). This suggests that health interventions, as designed and implemented by occupational health nurses, need to consider targeted interventions with specific groups.

Ergonomic design and adaptation is a key issue in such intervention planning (Larrea-Araujo et al., 2021). The findings of the review highlight the importance of designing ergonomic work environments to mitigate the negative effects of WFH (Larrea-Araujo et al., 2021). In this regard, occupational health nurses will best serve employees by expanding their skill set in relation to ergonomic design and mitigations (with greater understanding of the inter-relationship of home furnishing, lighting and ventilation with optimal health promoting at home workspace design) and how to encourage workers to adapt their homes in relation to these factors.

Similarly, there is a range of negative impacts associated with using online tools, which include “zoom fatigue” and vocal tract discomfort (Kenny, 2020; Shockley et al., 2021). To address these impacts, occupational health nurses will want to promote a range of harm reduction practices such as switching off the camera and microphone when possible (Bennett et al., 2021; Shockley et al., 2021). Several studies suggested benefits in terms of overall well-being linked to WFH (Björndell & Premberg, 2021; Dhont et al., 2020). Associated with these results were perceived job autonomy, job crafting, and a positive work schedule (Ingusci et al., 2021; Reizer et al., 2021; Wang et al., 2021)—all of which are associated with the concept “flow.” These results would suggest, therefore, that occupational health nurses are in a position to help remote workers identify and utilize these factors to improve their WLF—indeed we would argue WLF should be incorporated into occupational health nurses’ education, training, and professional philosophy.

Closely related to a sense of well-being is the positive effect WFH can have on sleep (Hallman et al., 2021) though perceived stress can reduce sleep quality and may be dependent on the ability of workers to psychologically detach from work (Rhower et al., 2020). The relationship of sleep to well-being is closely related to employee work performance and job satisfaction and needs special consideration from a practice viewpoint (Deng et al., 2022; Kun & Gadanecz, 2022).

Quality of sleep is also related to physical activity. Access to green and blue spaces and having pets are mediating factors related to physical activity among remote workers. Occupational health nurses are able to encourage remote workers to engage in physical activity and consider the resources available to them, such as access to outdoor spaces and pets, which are thought to promote a healthy lifestyle (Astell-Burt & Feng, 2021; Fukushima et al., 2021 Hoffman, 2021).

Overall, these results suggest that occupational health nurses’ roles should extend to promoting workers’ quality of life through a holistic approach to occupational health assessment and planning that goes beyond recommendations about how often remote workers need to be physically active. This would mean that occupational health nurses would need to address remote workers’ ability to detach from work psychologically and physically to improve well-being and thereby increase work satisfaction that supports a positive work engagement. These are the most significant elements to be identified through this review as areas that need to be addressed by occupational health nurses.

The expansion of the occupational health nurse’s role to the home is not without controversy as this relates to the employee’s right to privacy and autonomy. However, this can be negotiated if employers adopt policies informed by decent work and WLF in which there is an emphasis on the promotion of well-being within remote working and the rights of individual workers to be supported through consultation and collaboration.

## Limitations

There are several limitations to this review. These include the heterogeneity of the studies, and that most studies were cross-sectional surveys. Furthermore, the short timeframe considered makes the main findings also limited in its scope. The low evaluation of the retrieved studies implies the conclusions should be cautious.

## Implications for Practice

The rise of remote working means that occupational health nurses need to engage with a broader span of practice that should include the provision of advice in restructuring the home as a workspace and addressing specific physical and psychological challenges associated with home-based work and, within the precepts of WLF, engaging with the promotion of employee quality of life. This would include providing guidelines for employees on their personal work practices and their relationship to their well-being practices, such as when to take physical exercise and when to disengage with technology.

Adopting a holistic approach to manage challenges that did not exist within the boundaries of the traditional workspace means that occupational health nurses may also need to work collaboratively with other healthcare professionals, such as mental health practitioners, and general practitioners. By doing so, they will contribute to a more sustainable and productive remote work environment.

## References

[bibr1-21650799231176397] Ahmed F. Kim S. Nowalk M. P. King J. P. VanWormer J. J. Gaglani M. Zimmerman R. K. Bear T. Jackson M. L. Jackson L. A. Martin E. Cheng C. Flannery B. Chung J. R. Uzicanin A. (2020). Paid leave and access to telework as work attendance determinants during acute respiratory illness, United States, 2017–2018. Emerging Infectious Diseases, 26(1), 26–33. 10.3201/eid2601.190743 31855145 PMC6924903

[bibr2-21650799231176397] American Board for Occupational Health Nurses. (2021). The career guide occupational health nursing profession.

[bibr3-21650799231176397] Anderson A. J. Kaplan S. A. Vega R. P. (2015). The impact of telework on emotional experience: When, and for whom, does telework improve daily affective well-being? European Journal of Work and Organizational Psychology, 24(6), 882–897. 10.1080/1359432X.2014.966086

[bibr4-21650799231176397] Anderson D. Kelliher C. (2020). Enforced remote working and the work-life interface during lockdown. Gender in Management: An International Journal, 35(7/8), 677–683. 10.3390/ijerph19073799

[bibr5-21650799231176397] Astell-Burt T. Feng X. (2021). Time for “Green” during COVID-19? Inequities in Green and Blue space access, visitation and felt benefits. International Journal of Environmental Research and Public Health, 18(5), Article 2757. 10.3390/ijerph18052757 PMC7967263 33803166

[bibr6-21650799231176397] Bennett A. A. Campion E. D. Keeler K. R. Keener S. K. (2021). Videoconference fatigue? Exploring changes in fatigue after videoconference meetings during COVID-19. The Journal of Applied Psychology, 106(3), 330–344. 10.1037/apl0000906 33871270

[bibr7-21650799231176397] Björndell C. Premberg Å . (2021). Physicians’ experiences of video consultation with patients at a public virtual primary care clinic: A qualitative interview study. Scandinavian Journal of Primary Health Care, 39(1), 67–76. 10.1080/02813432.2021.1882082 33650941 PMC7971243

[bibr8-21650799231176397] Chang Y. Chien C. Shen L. F. (2021). Telecommuting during the coronavirus pandemic: Future time orientation as a mediator between proactive coping and perceived work productivity in two cultural samples. Personality and Individual Differences, 171, Article 110508. 10.1016/j.paid.2020.110508 PMC7648512 33191964

[bibr9-21650799231176397] Chong S. Huang Y. Chang C. D. (2020). Supporting interdependent telework employees: A moderated-mediation model linking daily COVID-19 task setbacks to next-day work withdrawal. The Journal of Applied Psychology, 105(12), 1408–1422. 10.1037/apl0000843 33271029

[bibr10-21650799231176397] Collins A. M. Hislop D. Cartwright S. (2016). Social support in the workplace between teleworkers, office-based colleagues and supervisors. New Technology, Work and Employment, 31(2), 161–175. 10.1111/ntwe.12065

[bibr11-21650799231176397] Czakert J. P. Pais L. dos Santos Rebelo N. Ramos P. N. Scheibein F. Wells J. S. G. Berger R. (2022). Personal excellence based profiling to identify and apply tools and trainings for a better and sustainable work-life-flow. EDULEARN22 Proceedings, pp. 2365–2369. 10.21125/edulearn.2022.0613

[bibr12-21650799231176397] Darouei M. Pluut H. (2021). Work from home today for a better tomorrow! How working from home influences work-family conflict and employees’ start of the next workday. Stress and Health, 37(5), 986–999. 10.1002/smi.3053 33887802 PMC9291295

[bibr13-21650799231176397] Deng Y. Cherian J. Kuman K. Sarminah S. Abbas J. Sial M. S. Popp J. Olah J. (2022). Impact of sleep deprivation on job performance of working mothers: Mediating effect of workplace deviance. International Journal of Research in Public Health, 19(7), Article 3799. 10.3390/ijerph19073799 PMC8997657 35409482

[bibr14-21650799231176397] Department of Enterprise, Trade and Employment. (2021, August 20). Tánaiste publishes views on right to request remote work. Government of Ireland. https://www.gov.ie/en/press-release/f1b2d-tanaiste-publishes-views-on-right-to-request-remote-work/

[bibr15-21650799231176397] Dhont S. Derom E. Van Braeckel E. Depuydt P. Lambrecht B. N. (2020). The pathophysiology of “happy” hypoxemia in COVID-19. Respiratory Research, 21, Article 198. 10.1186/s12931-020-01462-5 PMC7385717 32723327

[bibr16-21650799231176397] dos Santos N. R. (2019). Decent work expressing universal values and respecting cultural diversity: Propositions for intervention. Psychologica, 62(1), 233–250. 10.14195/1647-8606_62-1_12

[bibr17-21650799231176397] Edwards L. Rutter G. Iverson L. Wilson L. Chadha T. S. Wilkinson P. Milojevic A. (2021). Personal exposure monitoring of PM2.5 among US diplomats in Kathmandu during the COVID-19 lockdown, March to June 2020. The Science of the Total Environment, 772, Article 144836. 10.1016/j.scitotenv.2020.144836 PMC7980227 33770893

[bibr18-21650799231176397] Estrada-Muñoz C. Vega-Muñoz A. Castillo D. Müller-Pérez S. Boada-Grau J. (2021). Technostress of Chilean Teachers in the Context of the COVID-19 Pandemic and Teleworking. International Journal of Environmental Research and Public Health, 18(10), Article 5458. https://doi.org/1010.3390/ijerph18105458 10.3390/ijerph18105458 PMC8160750 34065219

[bibr19-21650799231176397] Ferraro T. Pais L. dos Santos N. R. (2015). Decent work: An aim for all, made by all. International Journal of Social Sciences, IV, 3, 30–42. 10.20472/SS2015.4.3.003

[bibr20-21650799231176397] Ferraro T. Pais L. dos Santos N. R. Moreira J. M. (2018). The decent work questionnaire: Development and validation in two samples of knowledge workers. International Labour Review, 157(2), 243–265. 10.1111/ilr.12039

[bibr21-21650799231176397] Fisher K. A. Olson S. M. Tenforde M. W. Feldstein L. R. Lindsell C. J. Shapiro N. I. Files D. C. Gibbs K. W. Erickson H. L. Prekker M. E. Steingrub J. S. Exline M. C. Henning D. J. Wilson J. G. Brown S. M. Peltan I. D. Rice T. W. Hager D. N. Ginde A. A. . . . CDC COVID-19 Response Team. (2020). Telework before illness onset among symptomatic adults aged ≥18 years with and without COVID-19 in 11 outpatient health care facilities—United States, July 2020. MMWR. Morbidity and Mortality Weekly Report, 69(44), 1648–1653. 10.15585/mmwr.mm6944a4 33151918 PMC7643895

[bibr22-21650799231176397] Fukushima N. Machida M. Kikuchi H. Amagasa S. Hayashi T. Odagiri Y. Takamiya T. Inoue S. (2021). Associations of working from home with occupational physical activity and sedentary behavior under the COVID-19 pandemic. Journal of Occupational Health, 63(1), Article e12212. 10.1002/1348-9585.12212 PMC7938758 33683779

[bibr23-21650799231176397] Gabaglio E. Jacobs G. Bonetti A. Plassman R. (2002). Framework agreement on telework (issue brief).

[bibr24-21650799231176397] Giovanis E. Ozdamar O. (2022). Implications of COVID-19: The effect of working from home on financial and mental well-being in the UK. International Journal of Health Policy and Management, 9(11), 1635–1641. 10.34172/ijhpm.2021.33 PMC9808217 33949816

[bibr25-21650799231176397] Gleason A. M. (2021). Remote monitoring of a work-from-home employee to identify stress: A case report. Workplace Health & Safety, 69(9), 419–422. 10.1177/2165079921997322 33880979

[bibr26-21650799231176397] Grawitch M. J. Gottschalk M. Munz D. C. (2006). The path to a healthy workplace: A critical review linking healthy workplace practices, employee well-being, and organizational improvements. Consulting Psychology Journal: Practice and Research, 58(3), 129–147. 10.1037/1065-9293.58.3.129

[bibr27-21650799231176397] Grech S. Borg J. N. Cuschieri S. (2022). Back pain: An aftermath of Covid-19 pandemic? A Malta perspective. Musculoskeletal Care, 20(1), 145–150. 10.1002/msc.1574 34092018 PMC8242888

[bibr28-21650799231176397] Guest D. (2002). Perspectives on the study of work-life balance. Social Science Information, 41(2), 255–279. 10.1177/0539018402041002005

[bibr29-21650799231176397] Hallman D. M. Januario L. B. Mathiassen S. E. Heiden M. Svensson S. Bergström G. (2021). Working from home during the COVID-19 outbreak in Sweden: Effects on 24-h time-use in office workers. BMC Public Health, 21, Article 528. 10.1186/s12889-021-10582-6 PMC7968563 33731066

[bibr30-21650799231176397] Hoffman C. L. (2021). The experience of teleworking with dogs and cats in the United States during COVID-19. Animals: An Open Access Journal from MDPI, 11(2), Article 268. 10.3390/ani11020268 PMC7912221 33494484

[bibr31-21650799231176397] Houle M. Lessard A. Marineau-Bélanger É. Lardon A. Marchand A.-A. Descarreaux M. Abboud J. (2021). Factors associated with headache and neck pain among telecommuters—A five days follow-up. BMC Public Health, 21, Article 1086. 10.1186/s12889-021-11144-6 PMC8179834 34090415

[bibr32-21650799231176397] Ingusci E. Signore F. Giancaspro M. L. Manuti A. Molino M. Russo V. Zito M. Cortese C. G. (2021). Workload, techno overload, and behavioral stress during COVID-19 emergency: The role of job crafting in remote workers. Frontiers in Psychology, 12, Article 655148. 10.3389/fpsyg.2021.655148 PMC8072041 33912116

[bibr33-21650799231176397] International Labour Organization. (2012). Decent work indicators: Concepts and definitions. https://www.ilo.org/integration/resources/pubs/WCMS_229374/lang–en/index.htm

[bibr34-21650799231176397] Izdebski Z. W. Mazur J. (2021). Changes in mental well-being of adult poles in the early period of the COVID-19 pandemic with reference to their occupational activity and remote work. International Journal of Occupational Medicine and Environmental Health, 34(2), 251–262. 10.13075/ijomeh.1896.01778 33734213

[bibr35-21650799231176397] Kawashima T. Nomura S. Tanoue Y. Yoneoka D. Eguchi A. Shi S. Miyata H. (2021). The relationship between fever rate and telework implementation as a social distancing measure against the COVID-19 pandemic in Japan. Public Health, 192, 12–14. 10.1016/j.puhe.2020.05.018 33607515 PMC7242969

[bibr36-21650799231176397] Kenny C. (2020). Dysphonia and vocal tract discomfort while working from home during COVID-19. Journal of Voice: Official Journal of the Voice Foundation, 36(6), Article 877. 10.1016/j.jvoice.2020.10.010 PMC7566822 33223124

[bibr37-21650799231176397] Kumar S. Kodidela S. Kumar A. Gerth K. Zhi K. (2020). Intervention and improved well-being of basic science researchers during the COVID 19 era: A case study. Frontiers in Psychology, 11, Article 574712. 10.3389/fpsyg.2020.574712 PMC7680890 33240163

[bibr38-21650799231176397] Kun A. Gadanecz P. (2022). Workplace happiness, well-being and their relationship with psychological capital: A study of Hungarian Teachers. Current Psychology, 41(1), 185–199. 10.1007/s12144-019-00550-0

[bibr39-21650799231176397] Lafferty A. Phillips D. Dowling-Hetherington L. Fahy M. Moloney B. Duffy C. Paul G. Fealy G. Kroll T. (2022). Colliding worlds: Family carers’ experiences of balancing work and care in Ireland during the COVID-19 pandemic. Health & Social Care in the Community, 30(3), 1133–1142. 10.1111/hsc.13365 33891356 PMC8251184

[bibr40-21650799231176397] Larrea-Araujo C. Ayala-Granja J. Vinueza-Cabezas A. Acosta-Vargas P. (2021). Ergonomic risk factors of teleworking in ecuador during the COVID-19 Pandemic: A cross-sectional study. International Journal of Environmental Research in Public Health, 18(10), Article 5063. 10.3390/ijerph18105063 PMC8151790 34064780

[bibr41-21650799231176397] Limbers C. A. McCollum C. Greenwood E. (2020). Physical activity moderates the association between parenting stress and quality of life in working mothers during the COVID-19 pandemic. Mental Health and Physical Activity, 19, Article 100358. 10.1016/j.mhpa.2020.100358 PMC7548083 33072187

[bibr42-21650799231176397] Loon M. Otaye-Ebede L. Stewart J. (2019). The paradox of employee psychological well-being practices: An integrative literature review and new directions for research. The International Journal of Human Resource Management, 30(1), 156–187. 10.1080/09585192.2018.1479877

[bibr43-21650799231176397] McDowell C. P. Herring M. P. Lansing J. Brower C. Meyer J. D. (2020). Working from home and job loss due to the COVID-19 pandemic are associated with greater time in sedentary behaviors. Frontiers in Public Health, 8, Article 597619. 10.3389/fpubh.2020.597619 PMC7674395 33224922

[bibr44-21650799231176397] Ng M. A. Naranjo A. Schlotzhauer A. E. Shoss M. K. Kartvelishvili N. Bartek M. Ingraham K. Rodriguez A. Schneider S. K. Silverlieb-Seltzer L. Silva C. (2021). Has the COVID-19 Pandemic Accelerated the Future of Work or Changed Its Course? Implications for Research and Practice. International Journal of Environmental Research and Public Health, 18, Article 10199. 10.3390/ijerph181910199 PMC8508142 34639499

[bibr45-21650799231176397] Oakman J. Kinsman N. Stuckey R. Graham M. Weale V. (2020). A rapid review of mental and physical health effects of working at home: How do we optimise health? BMC Public Health, 20, 1–13. 10.1186/s12889-020-09875-z 33256652 PMC7703513

[bibr46-21650799231176397] Oksanen A. Oksa R. Savela N. Mantere E. Savolainen I. Kaakinen M. (2021). COVID-19 crisis and digital stressors at work: A longitudinal study on the finnish working population. Computers in Human Behavior, 122, Article 106853. 10.1016/j.chb.2021.106853 PMC8569509 34754137

[bibr47-21650799231176397] Parker L. D. (2020). The COVID-19 office in transition: Cost, efficiency and the social responsibility business case. Accounting, Auditing & Accountability Journal, 33(8), 1943–1967. 10.1108/AAAJ-06-2020-4609

[bibr48-21650799231176397] Pheng L. S. Chua B. (2019). Work–life balance and work–life interface. In Pheng L.S. Chua B. (eds) Work-life balance in construction (pp. 7–17). Springer.

[bibr49-21650799231176397] Philips S. (2020). Working through the pandemic: Accelerating the transition to remote working. Business Information Review, 37(3), 129–134. 10.1177/0266382120953087

[bibr50-21650799231176397] Reizer A. Geffen L. Koslowsky M. (2021). Life under the COVID-19 lockdown: On the relationship between intolerance of uncertainty and psychological distress. Psychological Trauma: Theory, Research, Practice and Policy, 13(4), 432–437. 10.1037/tra0001012 33539162

[bibr51-21650799231176397] Rohwer E. Kordsmeyer A. C. Harth V. Mache S. (2020). Boundarylessness and sleep quality among virtual team members—A pilot study from Germany. Journal of Occupational Medicine and Toxicology, 15, Article 30. 10.1186/s12995-020-00281-0 PMC7542699 33042208

[bibr52-21650799231176397] Shklarski L. Abrams A. Bakst E. (2021). Navigating changes in the physical and psychological spaces of psychotherapists during Covid-19: When home becomes the office. Practice Innovations, 6(1), 55–66. 10.1037/pri0000138

[bibr53-21650799231176397] Shockley K. M. Clark M. A. Dodd H. King E. B. (2021). Work-family strategies during COVID-19: Examining gender dynamics among dual-earner couples with young children. The Journal of Applied Psychology, 106(1), 15–28. 10.1037/apl0000857 33151705

[bibr54-21650799231176397] Solmi M. Estradé A. Thompson T. Agorastos A. Radua J. Cortese S. Dragioti E. Leisch F. Vancampfort D. Thygesen L. C. Aschauer H. Schloegelhofer M. Akimova E. Schneeberger A. Huber C. G. Hasler G. Conus P. Cuénod K. Q. D. von Känel R. . . . Correll C. U. (2022). The collaborative outcomes study on health and functioning during infection times in adults (COH-FIT-Adults): Design and methods of an international online survey targeting physical and mental health effects of the COVID-19 pandemic. Journal of Affective Disorders, 299, 393–407. 10.1016/j.jad.2021.07.048 34949568 PMC8288233

[bibr55-21650799231176397] Sull D. Sull C. Zweig B. (2022). Toxic culture is driving the great resignation. MIT Sloan Management Review, 63(2), 1–9.

[bibr56-21650799231176397] Tušl M. Brauchli R. Kerksieck P. Bauer G. F. (2021). Impact of the COVID-19 crisis on work and private life, mental well-being and self-rated health in German and Swiss employees: A cross-sectional online survey. BMC Public Health, 21(1), Article 741. 10.1186/s12889-021-10788-8 PMC8052554 33865354

[bibr57-21650799231176397] United Nations. (2015). Transforming our world: The 2030 agenda for sustainable development.

[bibr58-21650799231176397] van Niekerk R. L. van Gent M. M . (2021). Mental health and well-being of university staff during the coronavirus disease 2019 levels 4 and 5 lockdown in an Eastern Cape university, South Africa. The South African Journal of Psychiatry, 27, Article 1589. 10.4102/sajpsychiatry.V27i0.1589 PMC8008007 33824757

[bibr59-21650799231176397] Van Zoonen W. Sivunen A. Rice R. E . (2020). Boundary communication: How smartphone use after hours is associated with work-life conflict and organizational identification. Journal of Applied Communication Research, 48(3), 372–392. 10.1080/00909882.2020.1755050

[bibr60-21650799231176397] Vyas L. Butakhieo N. (2021). The impact of working from home during COVID-19 on work and life domains: An exploratory study on Hong Kong. Policy Design and Practice, 4(1), 59–76. 10.1080/25741292.2020.1863560

[bibr61-21650799231176397] Wang B. Liu Y. Qian J. Parker S. K. (2021). Achieving effective remote working during the COVID-19 Pandemic: A work design perspective. Applied Psychology, 70(1), 16–59. 10.1111/apps.12290 33230359 PMC7675760

[bibr62-21650799231176397] World Health Organization. (2001). The role of the occupational health nurse in workplace health management. WHO Regional Office for Europe.

